# Spatial and space-time clustering and demographic characteristics of human nontyphoidal *Salmonella* infections with major serotypes in Toronto, Canada

**DOI:** 10.1371/journal.pone.0235291

**Published:** 2020-07-01

**Authors:** Csaba Varga, Patience John, Martin Cooke, Shannon E. Majowicz

**Affiliations:** 1 Department of Pathobiology, College of Veterinary Medicine, University of Illinois at Urbana-Champaign, Urbana, Illinois, United States of America; 2 School of Public Health and Health Systems, University of Waterloo, Waterloo, Ontario, Canada; 3 Department of Sociology & Legal Studies, University of Waterloo, Waterloo, Ontario, Canada; University of Tennessee Knoxville, UNITED STATES

## Abstract

Nontyphoidal *Salmonella enterica* (NTS) causes a substantial health burden to human populations in Canada and worldwide. Exposure sources and demographic factors vary by location and can therefore have a major impact on salmonellosis clustering. We evaluated major NTS serotypes: *S*. Enteritidis (n = 620), *S*. Typhimurium (n = 150), *S*. Thompson (n = 138), and *S*. Heidelberg (n = 136) reported in the city of Toronto, Canada, between January 1, 2015, and December 31, 2017. Cases were analyzed at the forward sortation area (FSA)—level (an area indicated by the first three characters of the postal code). Serotype-specific global and local clustering of infections were evaluated using the Moran's I method. Spatial and space-time clusters were investigated using Poisson and multinomial scan statistic models. Case-case analyses using a multinomial logistic regression model was conducted to compare seasonal and demographic factors among the different serotypes. High infection rate FSAs clustered in the central region of Toronto for *S*. Enteritidis, in the south-central region for *S*. Typhimurium, in north-west region for *S*. Thompson, and in the south-east region for *S*. Heidelberg. The relative risk ratio of *S*. Enteritidis infections was significantly higher in cases who reported travel outside of Ontario. The relative risk ratio of infections was significantly higher in summer for *S*. Typhimurium, and in fall for *S*. Thompson. The relative risk ratio of infection was highest for the 0–9 age group for *S*. Typhimurium, and the 20–39 age group for *S*. Heidelberg. Our study will aid public health stakeholders in designing serotype-specific geographically targeted disease prevention programs.

## Introduction

Nontyphoidal *Salmonella enterica* (NTS) is responsible for an estimated 150,716 illnesses, 1,565 hospitalizations, and 29 deaths each year in Canada, of which the majority are foodborne [1]. On a worldwide level, diarrheal and invasive infections caused by NTS have the highest yearly disease burden among all foodborne infections, with 4.07 million attributable Disability Adjusted Life Years [2]. Among the more than 2,600 currently known NTS serotypes, only a small portion cause human infection, most commonly gastroenteritis [3, 4]. In Canada, NTS serotype Enteritidis (*S*. Enteritidis), Typhimurium (*S*. Typhimurium), and Heidelberg (*S*. Heidelberg) are the most frequent serotypes, consisting of over 50% of all serotypes detected [5]. Despite constant attempts to decrease the incidence of NTS infections during the last decade, public health authorities have not been successful, due, in part, to the significant increase in infections with certain serotypes, such as *S*. Enteritidis [3, 4, 6].

Salmonellosis is a complex disease with a multitude of factors that influence its morbidity and mortality. Specific serotypes are likely to be linked to particular transmission routes based on their role in natural ecosystems [3]. Previous studies have described common food exposure sources for NTS infections with different serotypes. *Salmonella* Enteritidis was most commonly associated with the consumption of chicken, eggs, turkey and sprouts, *S*. Typhimurium with beef, dairy, pork and vegetables, and *S*. Newport with fruits and vegetables [7]. While the majority of NTS infections are foodborne, contact with infected animals or their contaminated environments should not be overlooked as an infection source since NTS are frequently carried asymptomatically by food animals or pets [8].

Studying infectious diseases in cities is increasingly important [9], considering the growth of urban populations, which are expected to exceed 60% of the total global population by 2050 [10]. Our study was conducted in the city of Toronto, the capital of the province of Ontario and the largest city in Canada, situated along Lake Ontario’s northwestern coast.

According to the 2016 Census, Toronto had a population of 2,731,571 [11]. Toronto’s neighborhoods are diverse, and the spatial heterogeneity of salmonellosis rates may occur from variations in underlying local socioeconomic, demographic and environmental risk factors [12, 13]. Identifying geographical areas with significantly higher or lower serotype-specific infection rates can offer valuable etiological indicators to guide the development of public health prevention and control programs to reduce the health burden of NTS infections [14].

Recent studies have applied a number of spatial epidemiological methods to identify spatial clusters of NTS infections with various serotypes, including *S*. 1,4,[5],12:i:- in Portugal [15], *S*. Enteritidis in Canada [13, 14], *S*. Napoli in Italy [16], and *S*. Wangata and *S*. Typhimurium in, Australia [17].

With the expansion of technological capabilities in geographic information systems (GIS) and disease mapping along with the growing power of applied spatial epidemiological methods, several recent studies have used these methods to understand the spread and area-level risk factors of various infectious diseases [18], including granulocytic ehrlichiosis [19], COVID-19 [20] and chlamydiosis [21] in the United States, chikungunya and dengue fever in Colombia [22], and dengue fever in Panama [23].

Case-case studies have been effectively used previously in outbreak investigations to identify specific exposure sources and demographic indicators of NTS serotype-specific infections, including serotype Typhimurium in Spain [24], serotype Dublin in France [25], serotype Chester in six European countries [26], serotypes Newport, Javiana, and Mississippi in Tennessee, US [27], and serotypes Heidelberg and Typhimurium in Ontario, Canada [28].

Despite the multitude of spatial epidemiological and case-case studies that investigated NTS infections, there is a lack of studies investigating spatial clustering and demographic determinants of serotype-specific NTS infections in an urban setting in Canada and worldwide. Therefore, to better understand demographic factors and differences in spatial and temporal clustering among major NTS serotype infections in the city of Toronto, we developed a stepwise analytical framework that linked spatial exploratory and statistical methods with GIS to evaluate the distribution and spatial and temporal clustering of major serotypes (*S*. Enteritidis, *S*. Typhimurium, *S*. Thompson, and *S*. Heidelberg) of NTS infections. In addition, a case-case multinomial analysis was conducted to test whether seasonal and demographic factors differed by specific serotypes.

## Methods

### Data sources

Salmonellosis is a reportable disease in Ontario under the *Health Protection and Promotion Act* [29], and it is diagnosed by the isolation of *Salmonella enterica* from stool (most samples), rectal swabs, urine, blood, or any other sterile site. All *Salmonella* spp. isolates are sent to the public health laboratories for serotyping based on the Kauffmann-White method [30]. Public health unit personnel follow up on each reported *Salmonella enterica* case to provide case management, discover potential causes of disease and report all their findings to the Ontario Ministry of Health and Long-Term Care’s integrated Public Health Information System (iPHIS) [31]. For this study, data on reported NTS infections were acquired from the Ontario Ministry of Health and Long-Term Care and were extracted from the iPHIS database by Public Health Ontario on 2018/12/10. We obtained data related to *Salmonella* cases’ forward sortation area (FSA) (the first three digits of the postal code), disease onset date, serotype, age, sex, outbreak and travel history that were diagnosed in the city of Toronto between January 1, 2015, and December 31, 2017. The data were fully anonymized and stripped of direct identifiers such as client name, address, and age, before they were provided to the authors. Specifically, the cases were coded with unique case IDs and client IDs; the residential addresses were provided at the forward sortation area (FSA) and public health unit (PHU)—levels; the ages were aggregated to five-year age categories; and dates of disease onset were provided as months and years, rather than specific dates. We included in our study the top four NTS serotypes, *S*. Enteritidis (n = 620), *S*. Typhimurium (n = 150), *S*. Thompson (n = 138), and *S*. Heidelberg (n = 136).

This study was reviewed and received ethics clearance through a University of Waterloo Ethics Committee (ORE # 40133), who determined that informed consent was not required, since only de-identified secondary data was used for the research.

### Study setting

**Fig 1:**
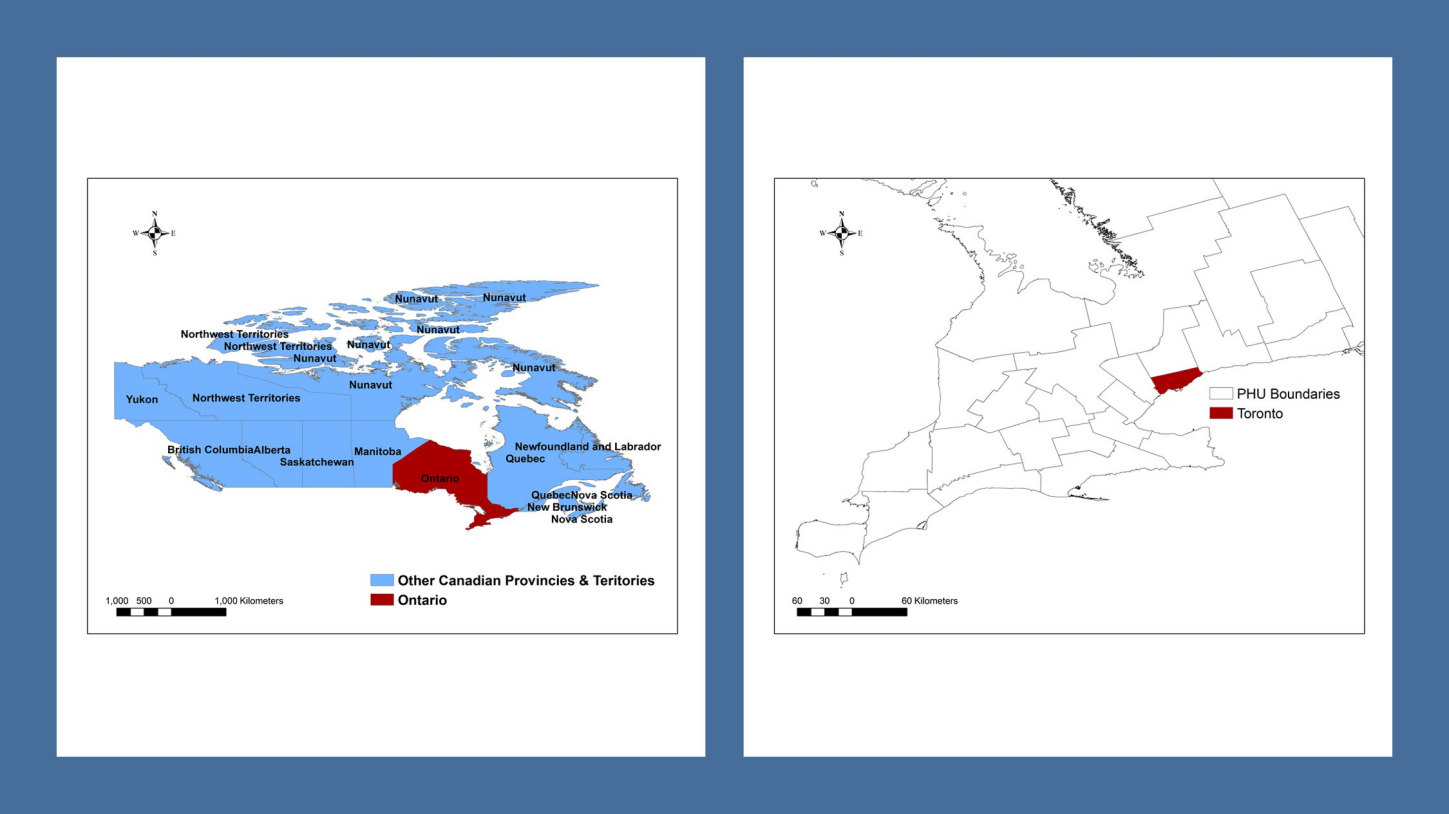
Map highlighting the location of the study area.

Our study involved the city of Toronto (Degrees, Minutes, Seconds (DMS) 43° 44′ 30″ North, 79° 22′ 24″ West), the capital of the province of Ontario, and the largest city in Canada, situated along Lake Ontario’s northwestern coast (Fig 1).

The analysis was conducted at the forward sortation area FSA-level, a well-defined geographical area represented by the first three characters of the postal code, designated by Canada Post Corporation, and based on the mail delivery regions of postal facilities. Population estimates and the cartographical boundary files for each FSA were obtained from the 2016 Census of Population [32, 33]. We excluded an FSA with less than 2,000 residents (n = 1) to obtain stable incidence rates. We included 95 FSAs containing a combined population of 2,730,089 (99.95% of the population). The annual population sizes of the 95 FSAs ranged from 2,951 to 75,897, with a mean of 28,737. Toronto covers an area of 630.2 square kilometers (243.33 square miles), with a maximum north-south distance of 21 kilometres, and a maximum east-west distance of 43 kilometers. Toronto FSAs were mapped using the City's commonly used projection, Universal Transverse Mercator (UTM), North American Datum (NAD) 1983, Zone 17 North.

### Disease mapping

Spatial heterogeneity of FSA-level NTS infection rates of the top four serotypes was evaluated and compared by following a stepwise analytical framework, which is described below.

Disease mapping and the global and local cluster analysis were performed using ArcGIS 10.7.1 (Environmental Systems Research Institute, Inc., Redlands, CA, USA).

All reported cases during the 3-year study period were geocoded and spatially joined to the FSA level. Incidence rates (IRs) per 100,000 persons for each serotype were calculated, by dividing the 3-year total number of cases per FSA by the 3-year total population estimates obtained from the 2016 Census of Population. Natural breaks that decrease the variance within classes and increase the variance between classes were used to classify IRs into five categories [34]. In addition, to account for unstable IRs of FSAs with small populations, we used Spatial Empirical Bayes method [35] with 2nd order queen contiguity weights to smooth these rates [36]. The queen criterion defines neighbors as spatial units sharing a common edge or a common vertex. In Toronto, where several irregularly shaped FSAs are present, using the queen criterion is recommended [37]. Distribution of non-smoothed and smoothed IRs of the four major NTS serotype infections across Toronto’s FSAs were illustrated by choropleth maps in ArcGIS 10.7.1.

### Spatial and space-time clustering analysis

#### Global and local Moran’s I

The Spatial Statistics Toolbox in ArcGIS 10.7.1 was used to evaluate global and local clustering of serotype specific NTS infection rates.

For the spatial statistical analysis, each FSA was represented by a polygon, its population-weighted centroid, and its serotype-specific infection rate. Euclidean distance bands were used to measure distances from each FSA centroid to neighboring FSA centroid. The “zone of indifference” conceptualization, a modified form of a distance decay parameter, was applied to account for zoning and edge effects [38]. This parameter gives maximum weights for the target FSA and all neighboring FSAs within a defined distance band, and once this distance is surpassed, neighboring FSAs are allocated smaller and smaller weights as the distance from the target FSA becomes greater. The null hypothesis for both global and local cluster analyses assumes a complete spatial randomness, i.e., that FSAs with high or low IRs are randomly distributed across the study area [38]. The null hypothesis is rejected when FSAs with high or low IRs are more spatially clustered than would be expected if the underlying spatial processes were truly random. When the null hypothesis is rejected, a Z-score and a p-value are given for the identified cluster.

Global clustering of serotype specific NTS IRs was evaluated using the global Moran’s I approach [39]. Several Euclidean distances with 1 km increments were manually selected and included in the model, starting with the 3.3 km distance band that was required for each FSA to have at least one neighbor. A large, positive Z-score (values ≥ 1.96), a statistically significant p value (p ≤ 0.05) and a positive Moran's I Index signified that FSA-level IRs clustered, while a large, negative Z-score (values ≤ -1.96) and a significant p-value and a negative Moran's I index signified that FSA-level IRs were dispersed in the study area [39]. For the local cluster analysis, we included the distance band that indicated maximum spatial clustering (highest Z-score) of IRs at the global clustering stage. If global spatial clustering was not significant, we used the 3.3 km distance band that was required for each FSA to have at least one neighbor.

Local Moran’s I statistics were used to detect local spatial clusters of FSA-level non-smoothed IRs during the study period [40].

The local Moran’s I statistic of spatial associations is given as:

$$I_{i}=\frac{x_{i}-\bar{X}}{S_{i}^{2}}\sum_{j=1,j\neq i}^{n}w_{ij}\;(x_{j}-\bar{X})$$ (1)

where $x_{i}$ is an attribute (i.e. infection rate) of future *i* (i.e. forward sortation area), $\bar{X}$ is the mean of the corresponding attribute, $w_{ij}$ is the spatial weight between future *i* and *j*, and:

$$S_{i}^{2}=\frac{\sum_{j=1,j\neq i}^{n}(x_{j}-\bar{X}{)}^{2}}{n-1}$$ (2)

With *n* equating to the total number of features.

The local Moran’s I statistic recognizes hot spots (i.e., high-high IR areas), cold spots (i.e., low-low IR areas) and outlier areas (high-low and low-high IR areas). A statistically significant positive Local Moran’s I Index indicates that the target FSA is surrounded by FSAs with identical IRs (high-high: FSA with a high rate enclosed by FSAs with high rates; low-low: FSA with a low rate surrounded by FSAs with low rates). A statistically significant negative local Moran’s I Index signifies that the target FSA is surrounded by FSAs with different IRs (high-low: FSA with a high rate surrounded by FSAs with low rates; low-high: FSA with a low rate surrounded by FSAs with high rates).

#### Scan statistic

The scan statistic [41] with SaTScan software version 9.6 was used to identify purely spatial and space-time clusters of serotype specific NTS infections. At the first step, we created separate models for *S*. Enteritidis, *S*. Typhimurium, *S*. Thompson, and *S*. Heidelberg infections, and used a retrospective discrete Poisson model, which assumes that the number of infections in each FSA are Poisson-distributed, based on a known background population [42]. At the second step we built a multinomial model, in which each outcome was a salmonellosis case, with each case belonging to one of the four *Salmonella* serotypes, Enteritidis, Typhimurium, Thompson, and Heidelberg. The multinomial scan statistic concurrently looks for high or low clusters of any of the four serotypes, adjusting for the overall geographical distribution of salmonellosis, and accounting for the multiple comparisons among the four serotypes when calculating the p-values [43].

Latitude and longitude Cartesian coordinates for each FSA centroid were calculated in ArcGIS 10.7.1. The smallest temporal and spatial units were the month of infection onset and the centroid of an FSA, respectively. Only high IR clusters were examined. In the space-time analysis we used a cylinder with a circular spatial base and height relating to time to identify high IR clusters [44]. Circular scanning window was selected to detect large, compact clusters. The scanning window moves across simultaneously in space and time comparing the rate of infections inside of the scanning window to the rate of infections outside of the scanning window using a Monte Carlo hypothesis testing with 999 replications [18]. If the infection rate is significantly (p-value ≤0.05) higher inside the window a relative risk is estimated, and a high rate cluster is identified. Salmonellosis is mainly a foodborne disease, and the contaminated food sources could be widespread; therefore, we set the scanning window to include up to 50% of the study population and up to 50% of the study period, to detect large primary and secondary clusters.

To circumvent the assumption that the relative risk of salmonellosis is similar throughout a significant spatial or space-time cluster, we also present and illustrate the relative risk for each FSA that belonged to a significant cluster. The relative risk (RR) for each FSA belonging to a cluster is obtained from Eq (3):

$$RR=\frac{c/e}{\left(C-c)/(C-e\right)}$$ (3)

Where c is the total number of salmonellosis cases in an FSA, e is the total number of expected cases in an FSA, and C is the total number of observed cases in Toronto. The RR is the estimated risk within an FSA divided by the risk outside of the FSA. If the RR is greater than 1, salmonellosis cases within that FSA have a higher risk to have salmonellosis than cases from all the other FSAs.

Statistically significant space and space-time clusters were visualized in ArcGIS 10.7.1. The outbreak history of cases that were included in statistically significant space-time clusters were assessed to evaluate if local outbreaks explained the clustering of cases.

### Case-case multinomial logistic regression analysis

To compare individual-level demographic characteristics and seasonal factors among different NTS serotypes a case-case analysis [45, 46] was conducted. A multinomial logistic regression model was built where the outcome variable had four categories representing salmonellosis infections caused by serotypes Enteritidis, Typhimurium, Thompson, and Heidelberg. Serotype Enteritidis was included in the model as the base category to which the other three serotypes were compared.

The predictor variables were year, season, age group, sex, outbreak status, and travel history. Date of disease onset was used to assign each case to a specific year and season. The categorical year variable represented a consecutive 12-month period from January 1st to December 31st; therefore, there were three groups (2015, 2016, and 2017). The categorical season variable consisted of winter (December, January, and February), spring (March, April, and May), summer (June, July, and August), and fall (September, October, and November). The categorical age variable was divided into five categories: cases aged between 0 and 9, 10 and 19, 20 and 39, 40 and 59, and cases 60 years of age and greater.

## Results

### Disease mapping

A total of 620 laboratory-confirmed nontyphoidal *Salmonella* serotype Enteritidis (184 cases in 2015, 191 in 2016, and 245 in 2017), 150 Typhimurium (55 cases in 2015, 47 in 2016, and 48 in 2017), 138 Thompson (56 cases in 2015, 67 in 2016, and 15 in 2017), and 136 Heidelberg (52 cases in 2015, 46 in 2016, and 38 in 2017) infections were reported in the city of Toronto during the three year study period.

**Fig 2:**
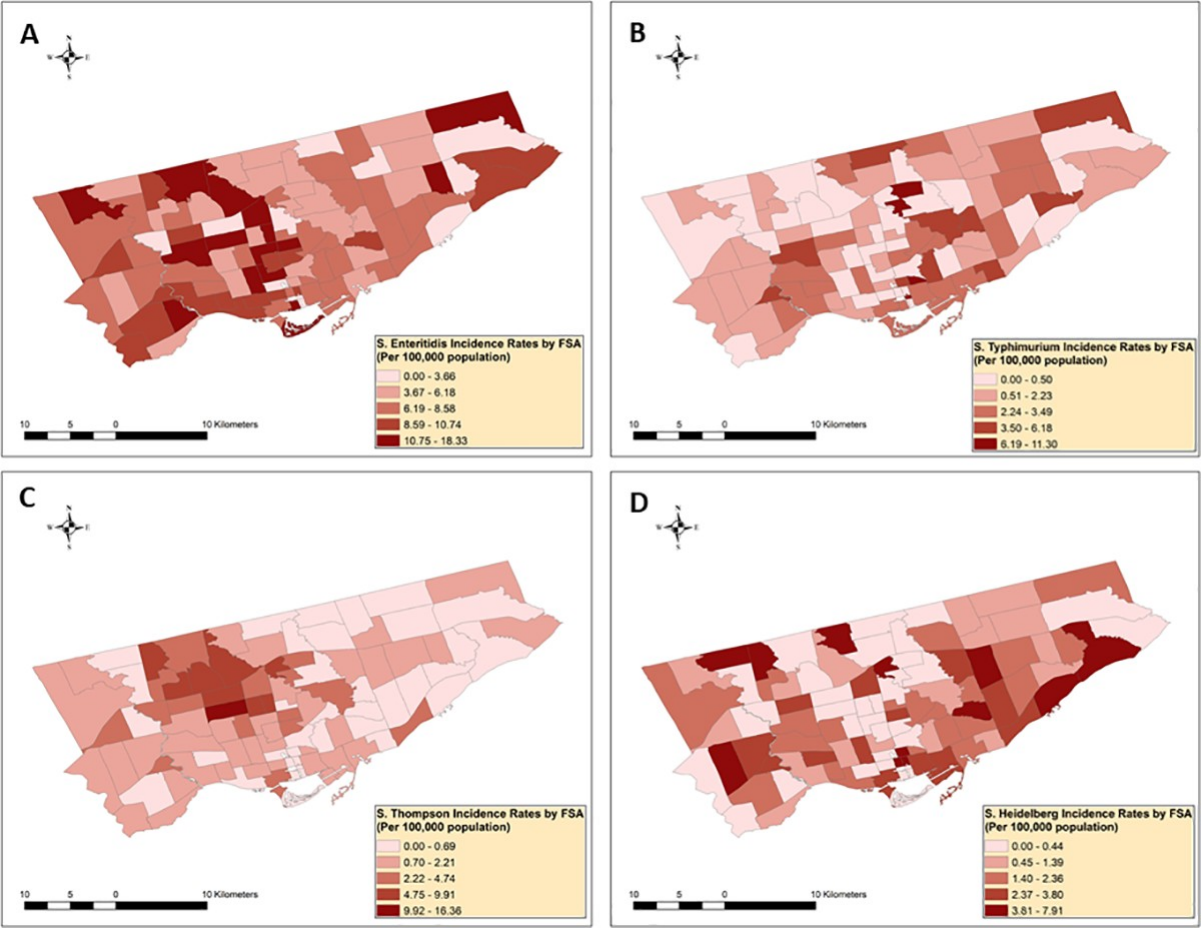
Distribution of non-smoothed nontyphoidal *Salmonella enterica* infection rates with serotypes (A) Enteritidis (n = 620), (B) Typhimurium (n = 150), (C) Thompson (n = 138) and (D) Heidelberg (n = 136) in Toronto’s forward sortation areas (n = 95), 2015−207^1^.

^1^Mean forward sortation area (FSA)-level incidence rates were obtained by dividing the FSA-level 3-year total number of cases by the 3-year total population estimates.The non-smoothed FSA-level mean serotype-specific IRs ranged from 0 to 18.33 (mean = 7.65) for Enteritidis, 0 to 11.30 (mean = 1.91) for Typhimurium, 0 to 16.36 (mean = 1.86) for Thompson, and 0 to 7.91 (mean = 1.65) for Heidelberg.

Fig 2 shows the non-smoothed IRs of NTS infections with the four main serotypes in each FSA.

**Fig 3:**
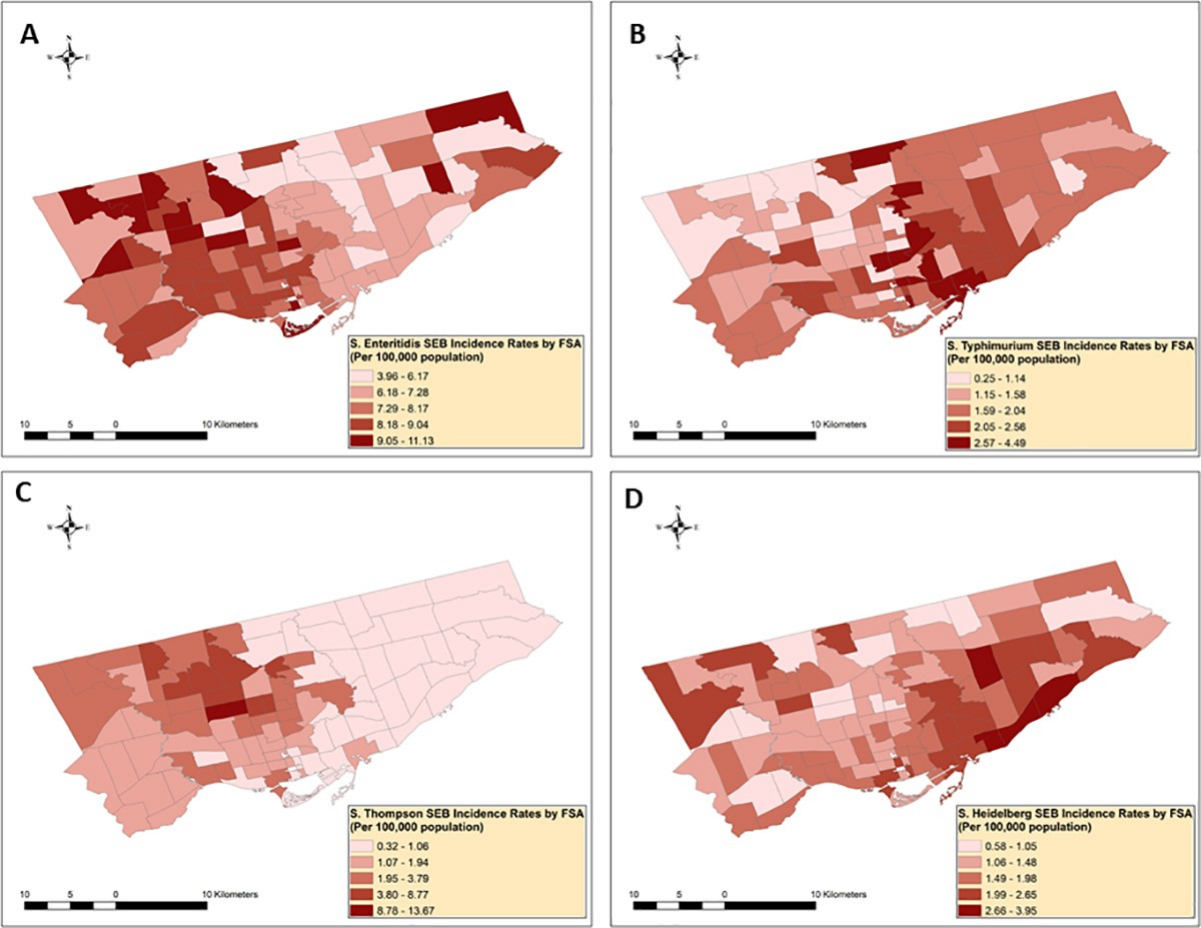
Distribution of smoothed nontyphoidal *Salmonella enterica* infection rates with serotypes (A) Enteritidis (n = 620), (B) Typhimurium (n = 150), (C) Thompson (n = 138) and (D) Heidelberg (n = 136) in Toronto’s forward sortation areas (n = 95), 2015−207^1, 2^. ^1^Spatial empirical Bayes smoothing method with 2nd order queen contiguity weights was used to smooth the mean forward sortation area (FSA)-level incidence rates.

Fig 3 shows the spatial empirical Bayes smoothed IRs of NTS infections with the four main serotypes.

The smoothed FSA-level IRs ranged from 3.96 to 11.13 (mean = 7.63) for Enteritidis, 0.25 to 4.49 (mean = 1.86) for Typhimurium, 0.32 to 13.67 (mean = 1.89) for Thompson, and 0.58 to 3.95 (mean = 1.63) for Heidelberg.

### Spatial and space-time clustering results

#### Global and local Moran’s I result

Only *S*. Thompson infections clustered globally in the city of Toronto. Statistically significant positive Z-scores (2.30–10.70) were detected between 3.3 km and 13.3 km. The largest statistically significant positive Z-score was observed at 6.3 km (Z = 10.7, p <0.001), signifying maximum global spatial clustering of FSAs with high *S*. Thompson infection rates at this distance band.

**Fig 4:**
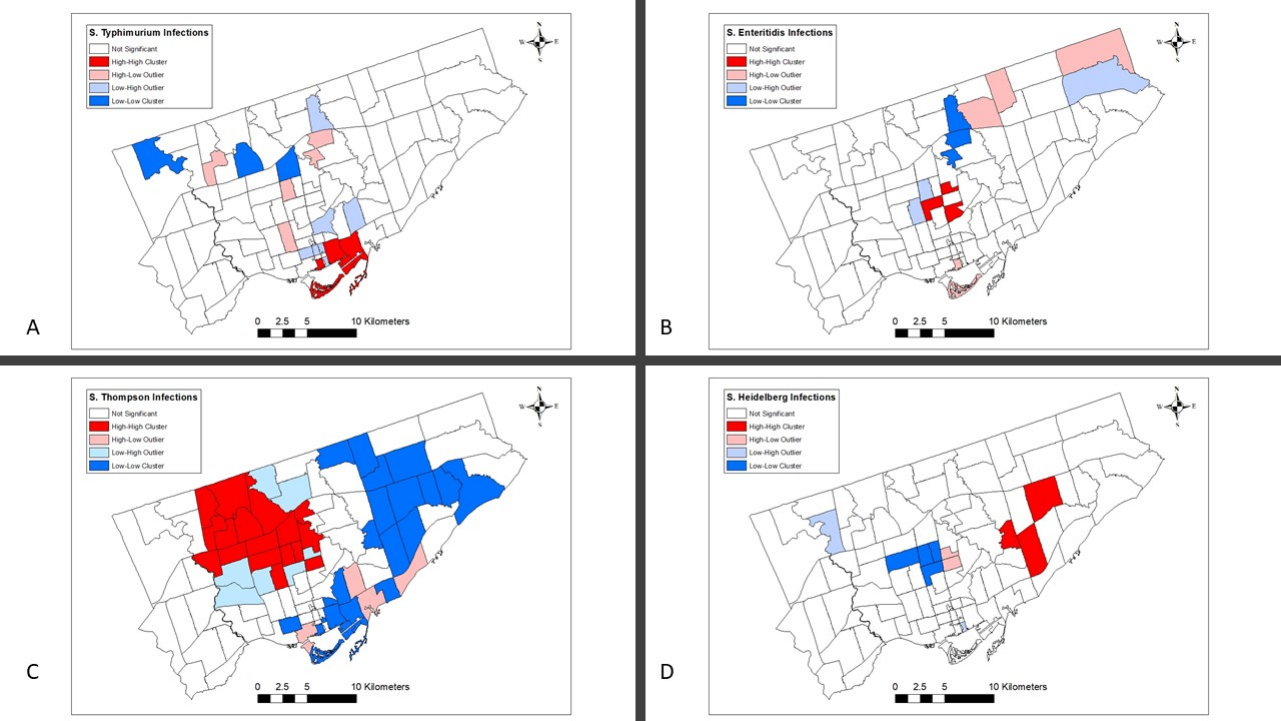
Local spatial clusters of nontyphoidal *Salmonella enterica* infections with serotypes (A) Enteritidis, (B) Typhimurium, (C) Thompson, and (D) Heidelberg in Toronto identified by the Moran’s I statistic.^1^Euclidean distance band of 3.3 km was used for serotype*s* Enteritids, Typhimurium and Heidelberg, and 6.3 km for serotype Thompson.

The local Moran’s I results are illustrated in Fig 4.

Three FSAs with High-High *S*. Enteritidis IRs, four FSA with High-Low IRs, two FSAs with Low-Low IRs, and three FSAs with Low-High were identified using the Local Moran’s I method (Fig 4, S1 Fig, S1 Table).

Three FSAs with High-High *S*. Typhimurium IRs, four FSA with High—Low IRs, three FSAs with Low-Low IRs, and seven FSAs with Low-High were identified using the local Moran’s I method (Fig 4, S1 Fig, S1 Table).

Seventeen FSAs with High-High *S*. Thompson IRs, four FSA with High-Low IRs, twenty FSAs with Low-Low IRs, and seven FSAs with Low-High were identified using the local Moran’s I method (Fig 4, S1 Fig, S1 Table).

Three FSAs with High-High *S*. Heidelberg IRs, two FSA with High-Low IRs, four FSAs with Low-Low IRs, and two FSAs with Low-High were identified using the Local Moran’s I method (Fig 4, S1 Fig, Fig 1, S1 Table).

#### Scan statistic

**Fig 5:**
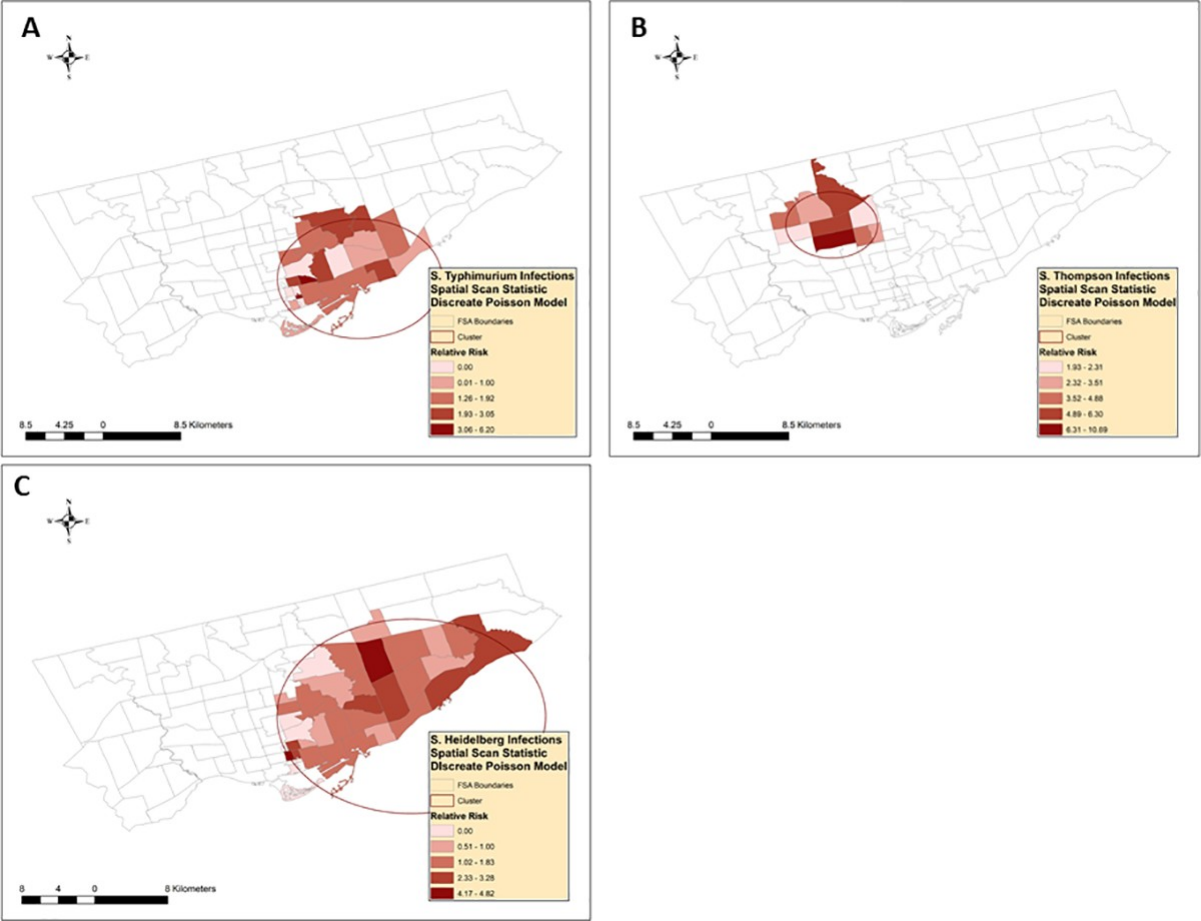
Spatial clusters of nontyphoidal *Salmonella enterica* infections with serotypes (A) Typhimurium, (B) Thompson, and (C) Heidelberg, identified by the scan statistic^1^. ^1^ Retrospective spatial analysis, scanning for clusters with high rates, using discrete Poisson models.

Three high rate purely spatial clusters were detected by using the discrete Poisson model (Fig 5, Table 1, S1 Fig, S1 Table).

**Table 1:** Spatial and space-time clusters of nontyphoidal *Salmonella enterica* infections with major serotypes in the city of Toronto, Ontario, Canada, 2015–2017^1^.

Serotype (N)	Model Type	Number of FSAs	Time Frame (Y/M)	Population	Number of cases	Expected cases	O/E	RR	P-value
Typhimurium (150)	Spatial	24	NA	529983	48	29.12	1.65	1.95	0.057
Thompson (138)	Spatial	9	NA	191219	48	9.67	4.97	7.08	< 0.001
Heidelberg (136)	Spatial	35	NA	934428	68	46.55	1.46	1.92	0.046
Typhimurium (150)	Space-Time	52	2015/7 to 2015/10	1264744	26	7.80	3.33	3.82	0.006
Thompson (138)	Space-Time	9	2016/9 to 2016/12	183772	30	1.03	29.01	36.79	< 0.001
Heidelberg (136)	Space-Time	12	2017/4 to 2017/5	374779	11	1.04	10.59	11.43	0.001

^1^ Results based on retrospective discrete Poisson models using the SaTScan™ software.

*Salmonella* Typimurium infections (n = 48) cases clustered in the central part of Toronto. Out of the 24 FSAs included in the cluster, 6 FSAs reported a relative risk of 0, one had a RR of 0.82, and one had a RR of 0.98. On the other hand, 16 FSA had a relative risk greater than one, ranging from 1.17 to 6.2.

*Salmonella* Thompson infections (n = 48) clustered in the north-central part of Toronto. All the FSAs included in the cluster have a relative risk greater than one, ranging from 1.93 to 10.69.

*Salmonella* Heidelberg infections (n = 68) clustered in the south-east part of Toronto. Out of the 35 FSAs included in the cluster, 5 FSAs reported a relative risk of 0, and 8 had a relative risk of one or less, which ranged from 0.51 to 1. On the other hand, 22 FSAs had a relative risk greater than one, ranging from 1.02 to 4.82.

**Fig 6:**
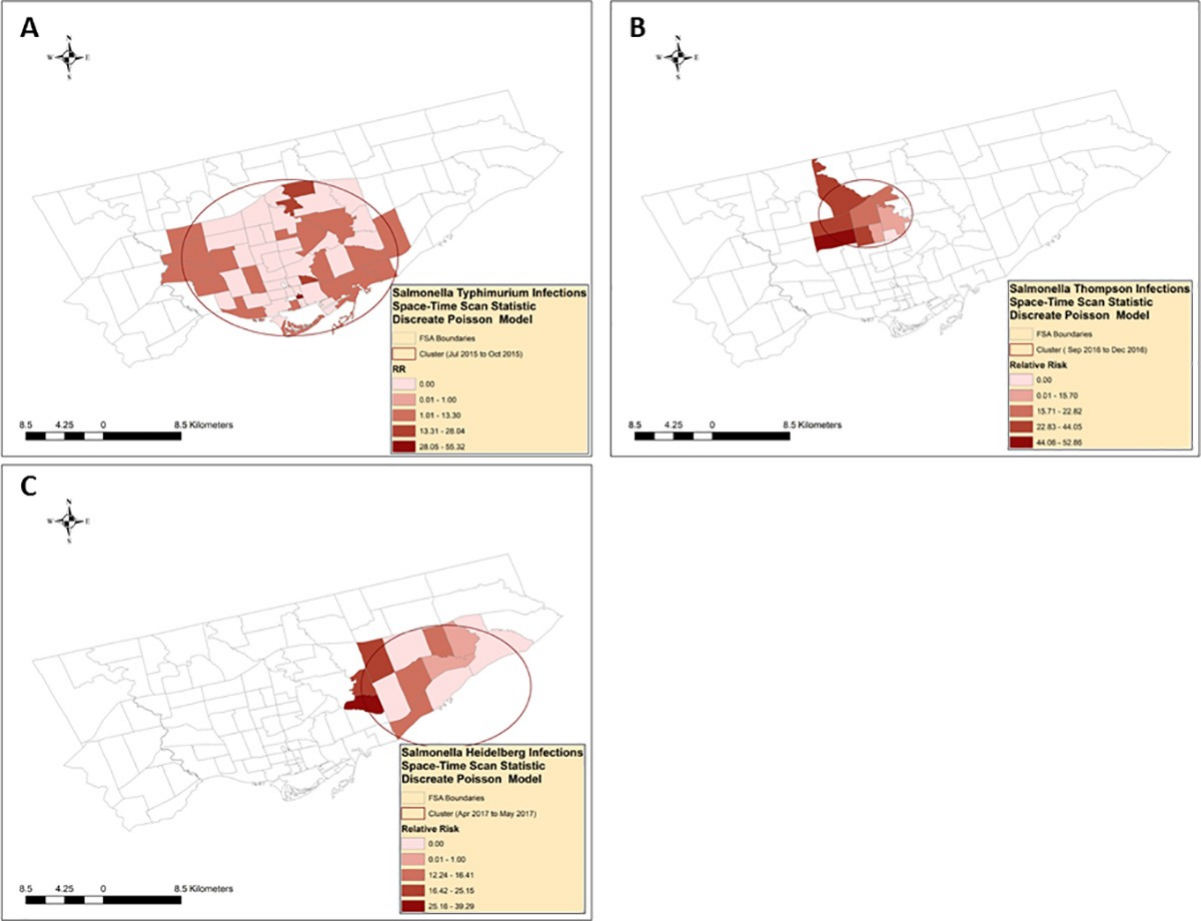
Space-time clusters of nontyphoidal *Salmonella enterica* infections with serotypes (A)Typhimurium, (B) Thompson, and (C) Heidelberg, identified by the scan statistic^1. 1^ Retrospective space-time analysis, scanning for clusters with high rates, using discrete Poisson models.

Three significant (p ≤ 0.05) high rate space-time clusters were detected (Table 1, Fig 6, S1 Fig, S1 Table).

One space-time cluster of *S*. Typhimurium infections (n = 26) occurred between July 2015 and October 2015 in the central part of Toronto (RR = 3.82). Out of the 52 FSAs included in the cluster, 32 FSAs had a relative risk of 0, and 20 FSAs had a relative risk greater than one, ranging from 3.48 to 55.32. A space-time cluster of *S*. Thompson infections (n = 30) occurred between September 2016 and December 2016 in the north-central part of Toronto (RR = 29.01). Out of the 9 FSAs included in the cluster, one FSA had a relative risk of 0, and 9 FSAs had a relative risk greater than one, ranging from 11.67 to 52.86.

A space-time cluster of *S*. Heidelberg infections (n = 11) occurred between April 2017 and May 2017 in the south-east part of Toronto (RR = 11.43). Out of the 12 FSAs included in the cluster, 4 FSAs have a relative risk of 0, and 8 FSAs have a relative risk greater than one, ranging from 9.89 to 39.29.

None of the 26 *S*. Typhimurium cases that were included in the space-time cluster were linked to an outbreak. Among the 30 *S*. Thompson cases that were included in the space-time cluster, 19 (63%) were linked to an outbreak. Of the 11 *S*. Heidelberg cases that were included in the space-time cluster, 8 cases (73%) were linked to an outbreak.

**Fig 7:**
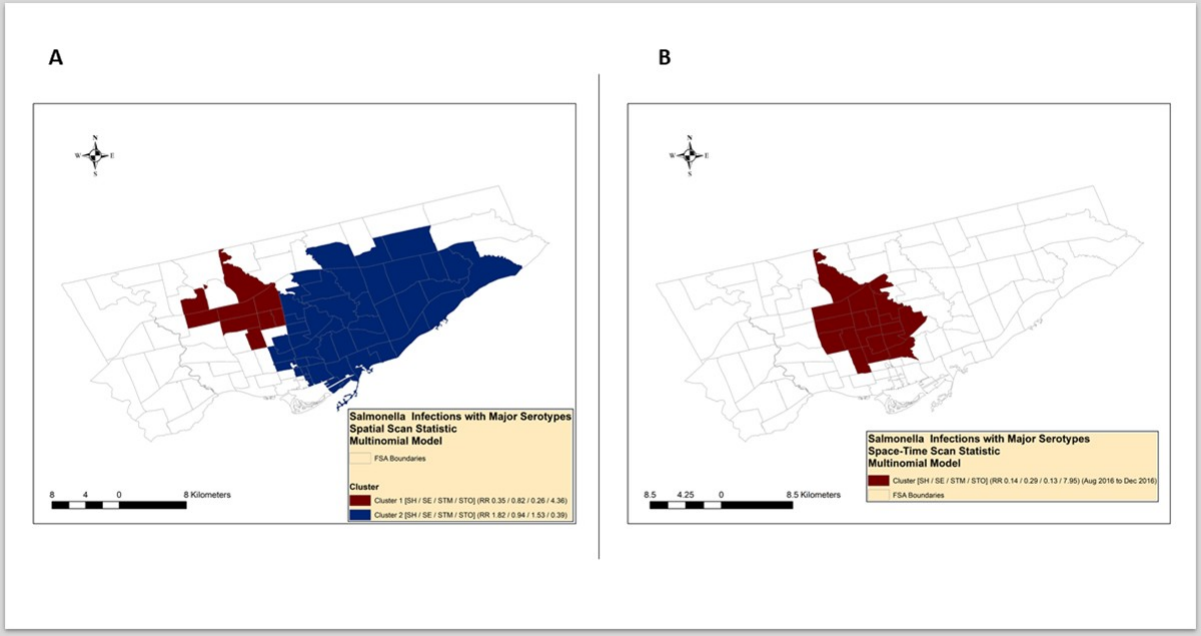
Spatial (A) and space-time (B) clusters of nontyphoidal *Salmonella enterica* infections with major serotypes identified by the multinomial scan statistic.

The purely spatial multinomial scan statistic identified two clusters (Fig 7, Table 2**)**. The primary spatial cluster was in the north-central part of Toronto, and included a relative risk greater than one for serotype Thompson and all the other serotypes had a smaller than one relative risk. The secondary spatial cluster was in the central and south-central part of Toronto and included a relative risk greater than one for serotypes Typhimurium and Heidelberg and had a relative risk smaller than one for serotypes Enteritidis and Thompson.

**Table 2:** Spatial and space time clusters of major nontyphoidal *Salmonella enterica* serotypes identified by the multinomial scan statistic in Toronto, Canada, 2015–2017^1^.

Model Type	Cluster	# FSAs	Time (M/Y)		Enteritidis	Typhimurium	Thompson	Heidelberg	P-Value
**Spatial**	C1	9	NA	Observed	60	5	50	6	< 0.001
				Expected	71.86	17.39	15.99	15.76	
				O/E	0.83	0.29	3.13	0.38	
				RR	0.82	0.26	4.33	0.35	
	C2	42	NA	Observed	236	75	28	74	< 0.001
				Expected	245.27	59.34	54.59	53.80	
				O/E	0.96	1.26	0.51	1.38	
				RR	0.94	1.53	0.39	1.82	
**Space-Time**	C1	19	2016/8 to 2016/12	Observed	9	1	40	1	0.001
				Expected	30.29	7.33	6.74	6.64	
				O/E	0.30	0.14	5.93	0.15	
				RR	0.29	0.13	7.95	0.14	

^1^ Retrospective spatial and space-time analysis, scanning for clusters with high rates, using multinomial models.

The space-time multinomial scan statistic identified one cluster (Fig 7, Table 2). The space-time cluster occurred between August 2016 and December 2016 in the north-central and central part of Toronto and included a relative risk greater than one for serotype Thompson and all the other serotypes had a smaller than one relative risk.

The primary cluster was in the north-central part of Toronto and included a relative risk greater than one for serotype Thompson and all the other serotypes have a smaller than one relative risk. The secondary cluster was in the central and south-central part of Toronto and included a relative risk greater than one for serotypes Typhimurium and Heidelberg and have a relative risk smaller than one for serotypes Enteritidis and Thompson.

### Case-case multinomial logistic regression analysis

The results of the multinomial logistic regression model of serotype-specific NTS infections are shown in Table 3.

**Table 3:** Demographic and seasonal factors for nontyphoidal *Salmonella enterica* infections with major serotypes in the city of Toronto, Ontario, Canada, 2015–2017^1^.

Outcome^2^	Predictor Variables		RRR^3^	95% CI^4^	P-value^5^
*S*. Typhimurium	Year	2015	Referent	-	-
		2016	0.89	0.53–1.48	0.65
		2017	0.68	0.41–1.13	0.14
	Season	Fall	Referent	-	-
		Winter	0.61	0.30–1.24	0.17
		Spring	0.59	0.31–1.13	0.11
		Summer	2.11	1.21–3.67	0.01
	Age (years)	0–9	Referent	-	-
		10–19	0.36	0.16–0.83	0.02
		20–39	0.53	0.31–0.90	0.02
		40–59	0.36	0.18–0.71	< 0.01
		≥ 60	0.76	0.39–1.47	0.41
	Sex (Male = 0, Female = 1)		0.99	0.65–1.51	0.95
	Travel		0.39	0.25–0.63	< 0.01
	Outbreak		1.60	0.72–3.58	0.25
*S*. Thompson	Year	2015	Referent	-	-
		2016	1.11	0.66–1.87	0.69
		2017	0.17	0.08–0.36	< 0.01
	Season	Fall	Referent	-	-
		Winter	0.44	0.22–0.85	0.01
		Spring	0.41	0.22–0.79	0.01
		Summer	0.35	0.18–0.68	< 0.01
	Age (years)	0–9	Referent	-	-
		10–19	0.64	0.56–3.09	0.52
		20–39	1.75	0.93–3.61	0.08
		40–59	1.31	0.78–3.56	0.19
		≥ 60	1.62	0.87–4.44	0.10
	Sex (Male = 0, Female = 1)		0.86	0.53–1.39	0.53
	Travel		0.12	0.06–0.24	< 0.01
	Outbreak		5.55	2.97–10.39	< 0.01
*S*. Heidelberg	Year	2015	Referent	-	-
		2016	0.88	0.52–1.50	0.65
		2017	0.52	0.30–0.90	0.02
	Season	Fall	Referent	-	-
		Winter	0.93	0.47–1.83	0.83
		Spring	1.18	0.63–2.20	0.60
		Summer	1.13	0.61–2.11	0.70
	Age (years)	0–9	Referent	-	-
		10–19	1.13	0.50–2.56	0.77
		20–39	1.93	1.03–3.61	0.04
		40–59	1.92	0.96–3.84	0.07
		≥ 60	1.99	0.95–4.17	0.07
	Sex (Male = 0, Female = 1)		0.88	0.56–1.38	0.58
	Travel		0.06	0.02–0.14	< 0.01
	Outbreak		1.12	0.51–2.46	0.78
*S*. Enteritidis	Base (referent) category				

^1^ Results of the multinomial logistic regression model.

^2^ Outcome: *S*. Enteritidis (N = 620) compared to *S*. Typhimurium (N = 150), *S*. Thompson (N = 138) and *S*. Heidelberg (N = 136).

^3^ RRR-Relative Risk Ratio.

^4^ CI-Confidence Interval of the RRR.

^5^ Statistically significant at P ≤ 0.05.

In the multinomial model, *S*. Typhimurium infections compared to *S*. Enteritidis infections, given the other variables in the model were held constant, the relative risk ratio was significantly higher in the summer (RRR = 2.11, 95% CI 1.21–3.67) compared to the fall season. Children 0–9 years of age (referent category), followed by individuals 20–39 years of age (RRR = 0.53, 95% CI 0.31–0.90), 10–19 years of age (RRR = 0.36, 95% CI 0.16–0.83) and 40–59 years of age (RRR = 0.36, 95% CI 0.18–0.71) had the highest risk of infection. Travel had a protective effect (RRR = 0.39, 95% CI 0.25–0.63) for *S*. Typhimurium infections. No significant differences were detected among years or between sexes.

*Salmonella* Thompson infections compared to *S*. Enteritidis infections, given the other variables in the model were held constant, the relative risk ratio was significantly lower in 2017 (RRR = 0.17, 95% CI 0.08–0.36) compared to 2015 (referent category). Compared to the fall season (referent category), the risk of infections were significantly lower in the winter (RRR = 0.44, 95% CI 0.22–0.85) spring (RRR = 0.41, 95% CI 0.22–0.79), and summer (OR = 0.35, 95% CI 0.18–0.68). Travel had a protective effect on *S*. Thompson infections (RRR = 0.12, 95% CI 0.06–0.24). *Salmonella* Thompson infections were significantly associated with outbreaks (RRR = 5.55, 2.97–10.39, 95% CI). No significant differences were detected among age groups or between sexes.

*Salmonella* Heidelberg infections compared to *S*. Enteritidis infections, given the other variables in the model were held constant, the relative risk ratio was significantly lower in 2017 (RRR = 0.52, 95% CI 0.30–0.90) compared to 2015 (referent category). Persons between 20 and 39 years of age (RRR = 1.93, 95% CI 1.03–3.61) had the highest risk of infection. *Salmonella* Heidelberg infections were significantly less likely to be travel related than *S*. Enteritidis infections (RRR = 0.06, 95% CI 0.02–0.14). No significant differences were detected among years, seasons, or between sexes.

## Discussion

Our study applied a stepwise analytical framework that linked spatial exploratory and statistical methods with GIS to assess and compare the distribution and spatial clustering of NTS infections with different serotypes across the city of Toronto. As the first exploratory spatial analytical step, we visually assessed the geographic extent of NTS infections with major serotypes across Toronto’s 95 FSAs by constructing disease choropleth maps. In cities with several areal units with small populations, the estimated infection rates become unreliable and unstable if several cases were detected in these regions [13]. To account for small area level unstable infection rates, we used spatial empirical Bayes method at the second step of our spatial exploratory analysis, which reduces the unbalanced infection rate estimates towards the local mean infection rate if local clustering is detected, and towards the global mean infection rate if no local clustering is found [47]. This method in our study reduced the highest infection rate estimates for each of the four NTS serotypes and helped us to focus our attention toward the overall spatial disease trends to evaluate visually FSAs of high and low IRs. Several high IR areas for each of the major NTS serotype infection were identified that were later recognized as statistically significant disease clusters.

We identified three space-time clusters across the city of Toronto. In our analysis, space-time clusters were defined as significantly higher than expected number of cases at a specific location and time period, which might or might not meet the definition of an outbreak [48]. Our space-time scan statistics correctly identified the *S*. Thompson and *S*. Heidelberg outbreaks as many of the cases that were included in the space-time clusters were linked to an outbreak by public health staff. However, none of the *S*. Typhimurium cases that were included in the space-time cluster were linked to an outbreak, suggesting that these cases did not have a common exposure source.

At the first spatial statistical step, we evaluated differences in global clustering among serotype-specific NTS infection IRs. Only *S*. Thompson IRs clustered globally across the city of Toronto. The extent of global clustering of *S*. Thompson IRs ranged from 3.3 km to 13.3 km, with a maximum global clustering at 6.3 km, suggesting that *S*. Thompson infections were widespread and involved several areas across Toronto. The local cluster analysis later supported this finding, by identifying a large high-high infection rate area in the northwest region of Toronto.

At the second spatial statistical step, we assessed and compared local clustering of serotype-specific NTS IRs by using the Moran’s I method. This method is ideal in areas with dissimilar IRs, because it not only identifies areas with high-high or low-low IRs, but also detects outlier areas where low IR areas are surrounded by high IR areas or high IR areas are surrounded by low IR areas. Interestingly, when we compared FSA-level high-high IR clusters among major NTS serotypes they differed in their size and location. The *S*. Thompson high-high IR cluster was the largest, including seventeen neighboring FSAs, in the northeast region of Toronto. The other NTS serotype clusters were small and included only three FSAs each, and were in the central region of Toronto for *S*. Enteritidis, in the northwest for *S*. Typhimurium, and the southeast for *S*. Heidelberg. The differences in serotype-specific geographical clustering among major NTS infections might be explained by differences related to their specific environmental, demographic, socioeconomic and host factors. Future targeted individual-level studies are needed in these areas to elucidate our study findings.

When we compared the spatial exploratory and spatial statistical methods for each NTS serotypes, the non-smoothed and smoothed maps visually identified areas with high and low rates, that were later identified by the local Moran’s I method as statistically significant high-high, low-low or outlier IR FSAs. In addition, the spatial and space-time scan statistic added extra information on the location and time frame of these clusters (Figs 2–7). The same high and low rate FSAs were consistently identified by each method, emphasizing the strength of our study methodology, and highlighting the consistency of FSA-level clustering of each NTS serotype.

We built multinomial scan statistic models to identify purely spatial and space-time clusters of nontyphoidal *Salmonella* infections with major serotypes. Multinomial scan statistic has some advantages over Poisson or binomial logistic regression models in that this model can investigate more than two outcomes (i.e. serotypes) concurrently in a single model. This approach was useful in our purely spatial analysis in detecting simultaneous excess risk ratios for serotypes Heidelberg and Typhimurium that might suggest a common exposure source (Fig 7, Table 2). The multinomial scan statistic is useful also in outbreak investigations, when a serotype compared to the “other” serotypes is strongly associated with a localized exposure source (i.e. local restaurant). This assumption was supported by our multinomial spatial and space-time scan statistic result that identified a cluster where only *S*. Thompson cases had an excess risk ratio (Fig 7, Table 2).

We identified a high *S*. Thompson infection rate space-time cluster in the north-east region of Toronto between September 2016 and December 2016 (Table 1, Fig 6), and 63% of cases that were included in this cluster were linked to an outbreak by public health enteric outbreak investigators. Interestingly, researchers from the neighbouring province of Quebec, Canada, reported an *S*. Thompson outbreak [49] at the same time period (November to December 2016) as the Toronto space-time cluster. The Quebec outbreak was linked to the consumption of chicken shawarma at a local restaurant chain in the city of Montreal and its neighboring regions. As our study did not have exposure history data, we cannot link the Montreal and Toronto outbreaks; however, it is possible that Toronto cases were exposed to the same food source, considering the probable inter-provincial poultry meat exchange through the supply chain, the identical time frame of illnesses, and the relatively rare serotype.

At the final step of our study, we performed a retrospective case-case multinomial analysis to test whether a specific serotype compared to all the other serotypes had different seasonal and demographic factors. Differences in annual infections among major NTS serotypes were identified. When compared to *S*. Enteritidis infections the relative risk ratio was higher in 2015 for *S*. Thompson and Heidelberg, and no differences among years were detected for *S*. Typhimurium. This finding is consistent with the results of previous Canadian [50] and US [51] studies that indicated an emergence in the number of *S*. Enteritidis infections during the last decade.

When we examined differences in cases’ age among major NTS serotypes we identified that the relative risk ratio of infections were highest in children 0–9 years of age for *S*. Typhimurium, in persons between 20 and 39 years of age for *S*. Thompson, in persons greater than 60 years old for *S*. Heidelberg. Differences in age predilection of various NTS serotypes might be explained by differences in their exposure sources and genetic determinants.

Our study detected a variation in NTS serotypes’ seasonal predilection. The relative risk ratio of infections was highest in the summer for *S*. Typhimurium, in the fall for *S*. Thompson, and no seasonal effect was detected for *S*. Heidelberg. Moreover, the relative risk ratio of cases who reported travel outside of Ontario was lowest among serotypes Typhimurium, Thompson, and Heidelberg when compared to *S*. Enteritidis infections. This finding agrees with previous studies conducted in Ontario, which showed that more than half of the *S*. Enteritidis infections detected in Ontario were travel-related [14, 52], and were linked to cases who visited resorts in the Caribbean and Mexico region during the winter and spring months [52].

Before extrapolating our study results several limitations should be mentioned. First, it is well documented that enteric surveillance systems commonly underestimate the true number of cases [53] and extrapolating our study results to the whole population requires caution. Also, in spatial epidemiological studies the Modifiable Areal Unit Problem (MAUP) should be considered where the chosen case aggregation scale and area boundary might affect the results of the analysis. Therefore, it is recommended to select an adequate scale that allows meaningful inferences about the spatial determinants of infectious diseases. We chose the smallest scale that is readily available in surveillance systems, the FSA, and believe that FSAs are adequately small scale to define areas with distinctive features. Besides, to account for differences in FSA size and spatial characteristics, we used the “zone of indifference” conceptualization parameter in our spatial statistical analysis.

For our scan statistic we used a circular scanning window that may miss irregularly shaped clusters. However, Toronto’s FSAs are compact, and irregularly shaped clusters are more common in elongated shaped areas (i.e. coastlines, rivers). In addition, we chose a relatively large window size that may include a single large cluster with several low infection rate areas. To overcome this problem, we calculated and illustrated the relative risks of each FSA that were included in each significant cluster.

Finally, our study was a spatial exploratory study that aimed to identify areas with high infection rates and seek to understand host factors for disease and did not aim to identify environmental exposure sources for NTS infections. Contamination of food products in retail stores and restaurants [54], differences in microbial quality of food among various neighborhoods [55], and food safety practices followed by food servers in local restaurants [56] have been described by previous studies as important factors for NTS infection outbreaks. Future studies should assess the role of local exposure sources, especially in high infection rate areas that were identified by our study.

## Conclusions

We demonstrated the utility of integrating geographic exploratory data visualization and spatial statistics analysis with GIS to identify and compare serotype-specific NTS infection clusters across Toronto’s FSAs. To investigate differences in seasonal and demographic factors among major NTS serotypes a case-case multinomial analytical method was performed. Several FSAs with high serotype-specific NTS rates were identified. Future studies in these FSAs should be conducted to identify local exposure sources and socioeconomic determinants for serotype specific NTS infections. A distinct seasonality of NTS infections were observed, the relative risk ratio of infections was highest in summer for *S*. Typhimurium and fall for *S*. Thompson. The relative risk ratio of infections was highest in young children for *S*. Typhimurium, in adults between 20 and 39 years of age for *S*. Thompson, and older adults for *S*. Heidelberg. The relative risk ratio of infections in cases who reported travel was highest among *S*. Enteritidis infections. Our research methodology could be applied to other enteric disease outbreak investigations across North America with a final goal to reduce the burden of NTS infections.

## Supporting information

S1 Fig For Figs 2–7. Toronto forward sortation area labels.
(TIF)

S1 Table For Figs 4–6. Forward sortation areas included in different local clusters.
(DOCX)
