# Supplementary figures and images for: Spatial and space-time clustering and demographic characteristics of human nontyphoidal Salmonella infections with major serotypes in Toronto, Canada
Source: PLoS One. 2020 Jul 1;15(7):e0235291. doi: 10.1371/journal.pone.0235291 (PMC7329108; doi:10.1371/journal.pone.0235291)

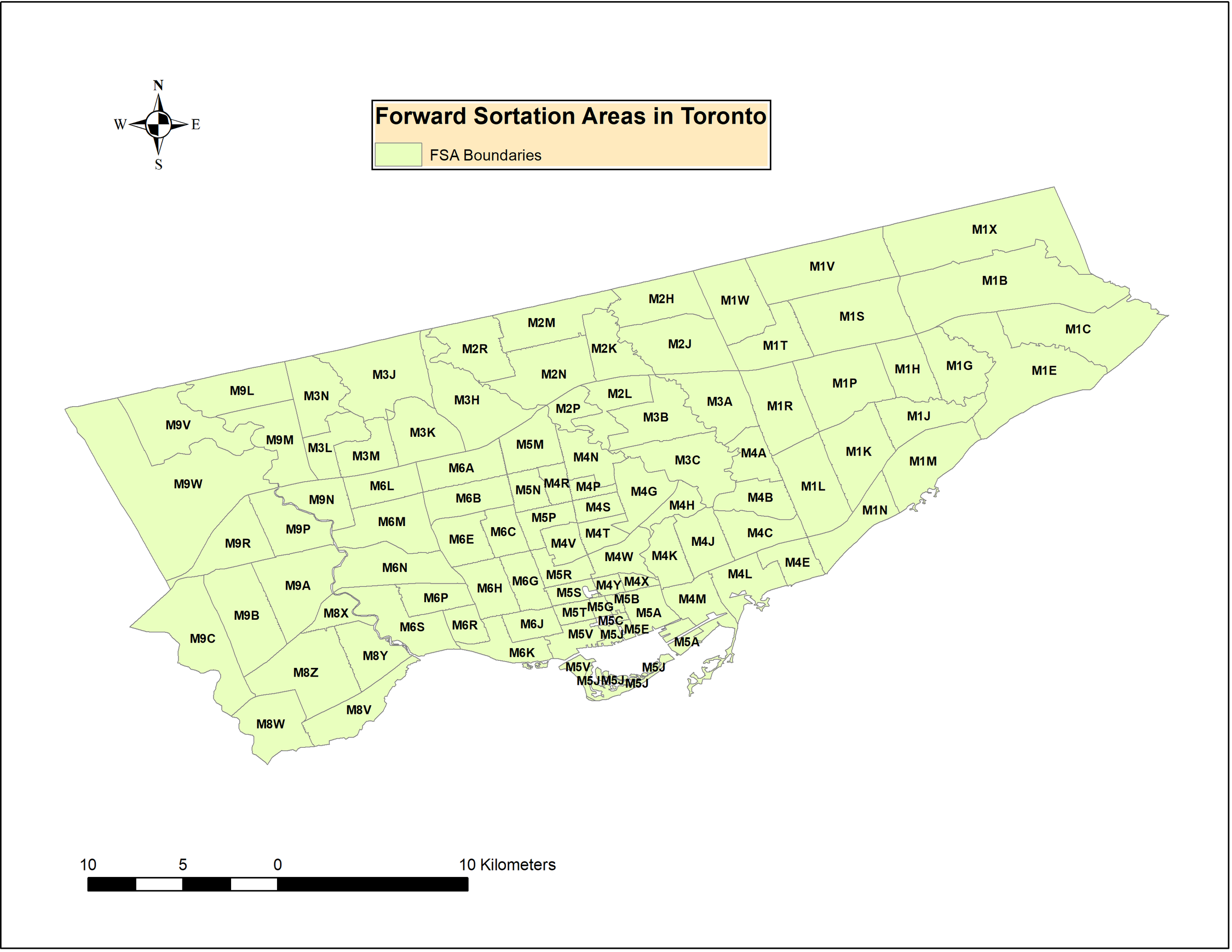

Supplement: S1 Fig — Toronto forward sortation area labels. (TIF) [file pone.0235291.s001.tif]
