# Supplementary material for: Spatial and space-time clustering and demographic characteristics of human nontyphoidal Salmonella infections with major serotypes in Toronto, Canada
Source: PLoS One. 2020 Jul 1;15(7):e0235291. doi: 10.1371/journal.pone.0235291 (PMC7329108; doi:10.1371/journal.pone.0235291)
Supplement: S1 Table — Forward sortation areas included in different local clusters. (DOCX) [file pone.0235291.s002.docx]

**S1 Table for Figs 4 - 6.** **Forward sortation areas included in different local clusters.**

| Serotype | Method | Type of Cluster | Forward Sortation Area |
| --- | --- | --- | --- |
| Enteritidis | Moran’s I | High-High | M4P, M4T, M5P |
|  |  | High-Low | M1W, M1X, M2J, M5J |
|  |  | Low-Low | M2K, M2L |
|  |  | Low-High | M1B, M5N, M6C |
| Typhimurium | Moran’s I | High-High | M4M, M5A, M5J |
|  |  | High-Low | M2L, M3L, M5N, M6G |
|  |  | Low-Low | M3K, M5M, M9V |
|  |  | Low-High | M2K, M4J, M4W, M5B, M5E, M5G, M5T |
|  | Discrete Poisson Scan | Spatial | M1L, M1N, M3C, M4A, M4B, M4C, M4E, M4G, M4H, M4J, M4K, M4L, M4M, M4T, M4W, M4X, M4Y, M5A, M5B, M5C, M5E, M5G, M5H, M5J |
|  |  | Space-Time | M1L, M2L, M2P, M3A, M3B, M3C, M4A, M4B, M4C, M4E, M4G, M4H, M4J, M4K, M4L, M4M, M4N, M4P, M4R, M4S, M4T, M4V, M4W, M4X, M4Y, M5A, M5B, M5C, M5E, M5G, M5H, M5J, M5M, M5N, M5P, M5R, M5S, M5T, M5V, M6A, M6B, M6C, M6E, M6G, M6H, M6J, M6K, M6L, M6M, M6N, M6P, M6R |
| Thompson | Moran’s I | High-High | M2P, M3H, M3J, M3K, M3L, M3M, M3N, M4N, M4R, M4S, M5M, M5N, M6A, M6B, M6C, M6L, M9N |
|  |  | High-Low | M1N, M4J, M4L, M5V |
|  |  | Low-Low | M1E, M1G, M1H, M1K, M1L, M1P, M1R, M1S, M1T, M1W, M2H, M4A, M4E, M4K, M4M, M4M, M4X, M5A, M5C, M5J |
|  |  | Low-High | M2N, M2R, M4P, M5P, M6E, M6M, M6N |
|  | Discrete Poisson Scan | Spatial | M3H, M3K, M3M, M4R, M5M, M5N, M6A, M6B, M6L |
|  |  | Space-Time | M1B, M1C, M1X, M2P, M3H, M4N, M4P, M4R, M5M, M5N, M6A, M6B |
| Heidelberg | Moran’s I | High-High | M1L, M1P, M4A |
|  |  | High-Low | M4P, M4S |
|  |  | Low-Low | M4R, M5N, M5P, M6B |
|  |  | Low-High | M5E, M9M |
|  | Discrete Poisson Scan | Spatial | M1N, M1L, M4E, M4C, M4B, M1K, M1M, M4L, M4A, M4J, M1J, M4H, M1R, M3C, M1P, M4K, M4M, M4G, M1H, M3A, M4W, M4X, M5A, M1G, M3B, M4T, M4S, M1T, M5B, M4Y, M5C, M4P, M1E, M5J, M5G |
|  |  | Space-Time | M1E, M1G, M1H, M1J, M1K, M1L, M1M, M1N, M1P, M1R, M4A, M4B |
